# Awareness and perspectives on patients' rights: a survey of health profession students at a Ugandan University

**DOI:** 10.4314/ahs.v25i4.22

**Published:** 2025-12

**Authors:** Nobert Olwete, Charles Ibingira, Erisa Sabakaki Mwaka

**Affiliations:** Department of Anatomy, School of Biomedical Sciences, College of Health Sciences, Makerere University

**Keywords:** Patients' rights, Health Profession students, Awareness, Perspectives, Bill of Rights

## Abstract

**Introduction:**

This study aimed to examine the awareness and perspectives of health professions students on patients' rights.

**Methods:**

A cross-sectional study was conducted among 294 undergraduate health profession students from three academic programs at a university in Uganda. Data were collected using a structured questionnaire. Descriptive statistics were used to summarize the data. Inferential analysis was performed using the Kruskall Wallis and Mann-Whitney U tests.

**Results:**

Most respondents were male (57%), with a mean age of 24 years (SD 4) and 57.1% were medical students. One hundred ninety-two students (65%) were aware of the Uganda Patients Charter and had mainly learned about it from class (46%) and online (20%). 63% agreed that a health provider may give medical treatment without the patient's informed consent. There were significant gender differences in the patients' right to impartial access to health care (p= 0.039) and voluntary participation in clinical training (p= 0.047). There were significant differences in perspectives on the patient's right to participate in care decisions and choose treatment plans across the three academic programs (p = 0.006).

**Conclusion:**

Most students were conversant with the different patient rights prescribed in the Uganda Patients Charter. However, they seemed not to appreciate the patient's right to informed consent in medical care.

## Background

The concept of human rights in patient care refers to the theoretical and practical application of general human rights principles to the patient care context, particularly in interactions between patients and healthcare providers^1^. It is universally acknowledged that all human beings possess individual and social rights, and certain groups within society are deemed particularly vulnerable and, therefore, require additional protections. Patients stand out as one of the most vulnerable, facing physical, psychological, social, and economic challenges^2^.

Patient rights are a fundamental human right, a quality assurance measure that protects patients against violation and discrimination and protects ethical practices^3^. In Uganda, patients' Bill of Rights (PBR) are included in the Uganda Patients Charter. A PBR is a set of claims that determine the status and needs of patients during the provision of health services and the obligations of the healthcare providers toward the patients as well as their relatives^4^. Patients' rights refer to specific legal privileges related to patients' physical, psychological, spiritual, and social needs that the healthcare system and the medical team observe^5^.

Although patients' rights are fundamental, it is only lately that they have gained prominence in Uganda^6^. In 2009, the Ministry of Health released the Uganda Patients' Charter^7^, which was recently amended and renamed the Patient Rights and Responsibility Charter^8^. The Patient Rights and Responsibility Charter aims to raise the standard of health care by empowering clients and patients to responsibly demand good quality health care from government facilities^7^. It also describes the rights and responsibilities of the patients and health workers. However, despite the availability of the Uganda Patients Charter, patients' rights are a poorly understood concept for patients and health care providers, including medical students in Uganda^6^. The intentions of the PBR are very noble, but its success greatly depends on healthcare providers' awareness and how well it is implemented.

Several studies have examined the awareness and practice of patient rights among health profession students. Al Anazi, Faraj^9^, in a study among medical students and interns in Saudia Arabia, reported an awareness of 69.3%. Another Saudi Arabian study reported an awareness of 65.5% of the PBR^4^. Conversely, some studies have reported low awareness of PBR, with many students unfamiliar with patient rights^2^,^10^. Some students have been reported to believe that patients' rights were ineffective^11^. Ghodsi and Hojjatoleslami^2^ reported low awareness among medical and paramedical students in various Iranian universities, with 47% of the respondents not familiar with PBR and only 16% having high awareness. A study from Uganda reported that almost 70% of healthcare workers at a national referral hospital have never heard about the Uganda Charter of Patients' Rights^6^. It is, therefore, evident that there are disparate levels of awareness and attitudes towards the PBR among health profession students. As future professionals, it is paramount that health profession students are well acquainted with patients' rights. This study, therefore, set out to investigate health profession students' awareness and perspectives on patients' rights to identify gaps in knowledge and enhance the education and training of future healthcare professionals.

## Methodology

### Study design

This was a cross-sectional survey that was conducted in April and May 2022 at Makerere University College of Health Sciences (MakCHS). Makerere University College of Health Sciences is a renowned hub for health research in Uganda and comprises five schools, including the Schools of Medicine, Health Sciences, Biomedical Sciences, Dentistry, and Public Health.

However, the study specifically targeted undergraduate students from the School of Medicine, Dentistry, and Health Sciences because they host students who participate in clinical activities and routinely interact with patients. Two hundred ninety-four undergraduate students enrolled in three academic programs-Bachelor of Medicine and Bachelor of Surgery (MBChB), Bachelor of Nursing (BSN), and Bachelor of Dental Surgery (BDS), were consecutively recruited using non-probability sampling. Bachelor of Dental Surgery and Bachelor of Medicine and Bachelor of Surgery are five-year programs whereas Bachelor of Nursing, is a four-year program. Bachelor of Dental Surgery, Bachelor of Medicine and Bachelor of Surgery commence clinical clerkship in Year 3 whereas Bachelor of Nursing commence in Year 2. All graduates from the three programs undergo mandatory internship for one year. Three students from the respective academic programs were employed as research assistants to assist in student recruitment and questionnaire distribution. Students were approached immediately after their lectures, and their written informed consent was obtained. Respondents were also requested to invite their colleagues. Respondents were given the opportunity to take the questionnaires home and complete them at leisure. The questionnaires were later collected by Research Assistants.

Data were collected using a structured survey instrument with closed-ended questions. There were different types of response categories, and these included True/False/Don't know, a 6-point Likert scale rating the level of agreement^12^, and categorical responses. The instrument covered three general topic areas, including demographic information, awareness of patients' rights, and perspectives of patients' rights. The survey tool was developed based on the Uganda Patients Charter^13^, a government policy that describes the rights and responsibilities of patients. It describes 19 patients' rights, five patients' responsibilities, and three health workers' responsibilities. This study was limited to patients' rights. Perspectives on patients' rights were determined using Likert-type questions. The instrument was pretested and adjusted accordingly to ensure that all questions were clear and understandable. Internal consistency and reliability were ensured by item analysis of the questionnaire during pretesting.

The survey was paper-based and was distributed by two research assistants. The sample size was calculated using the online OpenEpi software for sample size calculation for proportions (www.openepi.com) for a total student population of 632 (MBChB 470, BSN 66, BDS 96). We assumed that 50% of the students were aware of patient rights since there was no previous study among health profession undergraduate students, a precision of 5% (delta), and a design effect of 1. To allow for adequate power during subanalysis, a confidence level of 95%% for (1-β) was selected to give a sample size of 294.

Stratified non-random sampling was used for participant recruitment. Using openepi, the number of respondents to be recruited per academic program and year of study was calculated, as summarized in Table 1. Respondents were then recruited using convenience sampling.

**Table 1:** Sample size distribution per academic program and year of study

School	Eligible total population per academic program	Estimated sample size per academic program	Yr 2	Yr 3	Yr 4	Yr 5
MBChB	470	172	-	58	58	58
BDS	96	72	-	24	24	24
BNS	66	54	18	18	18	

Data were summarised using descriptive statistics. The Kruskall-Wallis test was used to analyze differences in perspectives on patients' rights across the different academic programs and academic years of study, as these variables had more than two categories. The Mann-Whitney U test was used to analyze gender differences in perspectives on patients' rights. Oneway analysis of variance (ANOVA) was used to investigate the differences between awareness of patients' rights and gender, academic program, and academic year of study. The level of significance was set at p<0.05.

## Results

Two hundred and ninety-four students participated in the study, of which 168 (57%) were male and 126 (43%) were female. The mean age was 24 years (SD 4, range 20- 40). Most respondents were medical students pursuing MB-ChB (57.1%). Participants' demographic information is summarised in Table 2.

**Table 2:** Demographic characteristics (n=294)

20-24	201	69.1
25-29	47	16.2
30+	43	14.8
Mean ± SD	24 ± 4	
**Gender**		
Male	168	57
Female	126	43
**Academic Program**		
Bachelor of Nursing	55	18.7
Bachelor of dental surgery	71	24.2
MBChB	168	57.1
**Year of study**		
Year 2	16	5.4
Year 3	102	34.7
Year 4	95	32.3
Year 5	81	27.6
**Religion**		
Catholic	103	35.5
Anglican	96	33.1
Pentecost	42	14.5
Islam	37	12.8
Other^1^	12	4.1

**Note:**

1 Adventist, Hindu, Jehovah's witness

### Awareness of the Patients' Bill of Rights

**Figure 1:**
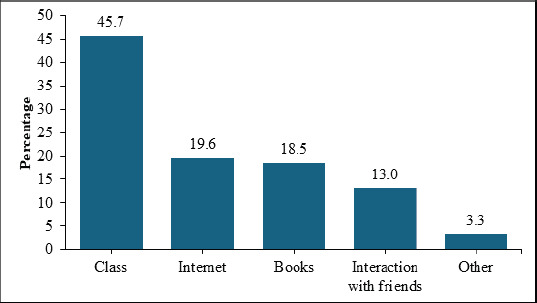
Means through which health profession students learned about the PBR

One hundred ninety-two respondents (65%) were aware of the PBR and had learned about it from multiple sources. Most respondents indicated that they had learned about the PBR from class, the Internet, and several other sources, as shown in Figure 1.

Religion was the only factor that significantly influenced respondents' awareness of the PBR (p= 0.03). Table 3 presents the association between the socio-demographic characteristics and awareness about the PBR.

**Table 3:** Association between selected socio-demographic characteristics and awareness about the PBR

	Awareness of patients' rights		
Variable	Aware Frequency (%)	Not aware Frequency (%)	p-value
**Gender**			0.86
Male	109 (56.7)	59 (57.8)	
Female	83 (43.2)	43 (42.2)	
**Academic program**			0.578
Bachelor of Nursing	38 (19.8)	17 (16.7)	
Bachelor of Dental Surgery	43 (22.4)	28 (27.5)	
MBChB	111 (57.8)	57 (55.9)	
**Year of study**			0.158
Year two	12 (6.3)	4 (3.9)	
Year three	59 (30.7)	43 (42.2)	
Year four	62 (32.3)	33 (32.4)	
Year five	59 (30.7)	22 (21.6)	
**Religion of student**			0.030*
Catholic	78 (41.3)	25 (24.8)	
Anglican	61 (32.3)	35 (34.7)	
Pentecost	25 (13.2)	17 (16.8)	
Islam	20 (10.6)	17 (16.8)	
Other^1^	5 (2.7)	7 (6.9)	

**Note:**

1 Adventist, Hindu, Jehovah's witness

* Significant at p<0.05

Respondents were asked about their awareness of the different patients' rights as outlined in the Uganda Patients' Charter of 2009. Overall, the number of patients' rights for which respondents provided correct responses ranged between four and 19, with a mean of 15.9 ± 2.2 (83.7%). Respondents showed low awareness of the right, “Patient physical or mental state does not permit obtaining his or her consent,” with only 24.8% providing the correct response. However, they demonstrated good awareness of all the other rights, as shown in Table 4. There was no significant difference in awareness of the PBR by gender (F= 0.07, p= 0.79), academic program (F=0.41, p= 0.66), year of study (F= 0.28, p= 0.84), and religion (F= 1.6, p= 0.18).

**Table 4:** Awareness of the different patients' rights as stipulated in the Uganda Patients' Charter

Awareness of specific patients' rights (n= 294)	True (Freq/percent)	False (Freq/percent)	Don't know (Freq/percent)
**Every person in need of medical care is entitled to impartial access to health care.**	269(91.8)*	18 (6.1)	6 (2.1)

**A patient has a right to appropriate medical available services irrespective of gender, age and religion.**	289(98.3)*	3 (1)	2 (0.7)
**A patient should not participate in decision making.**	25(8.5)	264 (90.1)*	4 (1.4)

**A patient should receive empathetic and respectful care.**	283(97.3)*	5 (1.7)	3 (1)
**A patients' participation in clinical trainings or programs should be voluntary, and informed consent shall be obtained and witnessed at all times**	276(93.9)*	11 (3.7)	7 (2.4)

**A patient has the right to privacy during a clinical examination.**	283(96.3)*	10 (3.4)	1 (0.3)
**A patient should not have a right to confidentiality.**	29(9.9)*	260 (88.4)	5 (1.7)

**A patient should not receive full explanation of his/her case.**	25 (8.6)	267 (91.4)*	0
**A patient should sign an informed consent form before any procedure.**	192(65.3)*	93 (31.6)	9 (3.1)

**A patient's physical or mental state does not permit obtaining his or her consent.**	73 (25)*	192 (65.8)	27 (9.3)
**A patient should not refuse to participate in any medical research.**	27 (9.2)	256 (87.1)*	11 (3.7)

**A patient should be treated in a hygienic environment.**	289(98.3)*	4 (1.4)	1 (0.3)
**A patient should be educated about financials costs and implications.**	284(96.6)*	10 (3.4)	0

**A patients should not refuse or discontinue treatment.**	38 (13)	248 (84.6)*	7 (2.4)
**Every should be referred for a second opinion with or without request.**	158(54.9)*	98 (34)	32 (11.1)

**A patient should not know the identifiable and professional position of a health care provider.**	42 (14.5)	241 (83.4)*	6 (2.1)
**A patient should not participate in care decision making.**	30 (10.2)	258 (87.8)*	6 (2)

**Every health facility should designate a person or a committeeto be responsible for patients' rights.**	283 (96.3)*	5 (1.7)	6 (2)

Perspectives on patients' rights. Respondents were asked for their level of agreement on the various patients' rights as outlined in the Uganda Patients' Charter. Most respondents strongly agreed or somewhat agreed with most of the patients' rights, as shown in Table 5. However, 185/293 (63.1%) of respondents strongly agreed or somewhat agreed that a health provider may give medical treatment without the patient's informed consent, and 217 (74.3%) decided that every person should be referred for a second opinion with or without request.

**Table 5:** Perspectives on patients' rights

Statement N= 293	Strongly agree	Somewhat agree	Somewhat disagree	Strongly disagree	Dont know
	Freq (%)	Freq (%)	Freq (%)	Freq (%)	Freq (%)
Every person in need of medical care is entitled to impartial access to health care.	224 (76.5)	46 (15.7)	7 (2.4)	13 (4.4)	3 (1)
The patient should be provided with appropriate medical services.	275 (93.9)	14 (4.8)	0	3 (1)	1 (0.3)
Patients should be represented in development of health policies.	152 (52.2)	87 (29.9)	17 (5.8)	25 (8.6)	10 (3.4)
Patients should receive empathetic and respectful care.	240 (81.9)	50 (17.1)	0	1 (0.3)	2 (0.7)
Patients' participation in clinical trainings and programs should be voluntary.	241 (82.3)	38 (13)	4 (1.4)	5 (1.7)	5 (1.7)
Patients should have a right to privacy during clinical examination.	271 (92.5)	17 (5.8)	3 (1)	0	2 (0.7)
Patients should have a right to confidentiality.	267 (91.1)	16 (5.5)	3 (1)	2 (0.7)	5 (1.7)
Patients should receive a full explanation of their cases and outcomes of care.	243 (82.9)	42 (14.3)	2 (0.7)	3 (1)	3 (1)
Patients or parents should sign informed consent forms before any medical procedure.	225 (77.1)	51 (17.5)	4 (1.4)	7 (2.4)	5 (1.7)
A health provider may give medical treatment without informed consent.	114 (39.3)	71 (24.5)	31 (10.7)	63 (21.7)	11 (3.8)
Patients can accept or refuse to participate in any medical research.	220 (75.6)	51 (17.5)	6 (2.1)	11 (3.8)	3 (1)
Patients should be treated in a hygienic environment.	272 (93.2)	13 (4.5)	1 (0.3)	5 (1.7)	1 (0.3)
Patients should be educated about precautions and financial costs.	247 (84.6)	35 (12)	3 (1)	4 (1.4)	3 (1)
Every person should be referred for a second opinion with or without request.	138 (47.3)	79 (27.1)	18 (6.2)	35 (12)	22 (7.5)
A patient has the right to refuse or discontinue treatment.	236 (80.6)	33 (11.3)	9 (3.1)	12 (4.1)	3 (1)
Patients should know the identity/name of the persons involved in health care.	227 (77.5)	48 (16.4)	9 (3.1)	7 (2.4)	2 (0.7)

This table presents participants' the level of agreement with the different patient's rights.

This table presents participants' the level of agreement with the different patient's rights. Differences in perspectives on patients' rights. There were significant gender differences in the rights to impartial access to health care (mean rank = 1.91 for males and 1.97 for females, p = 0.039), voluntary participation in clinical training and programs (mean rank = 1.95 for males and 1.99 for females, p = 0.047), and provision of medical treatment by a health provider without obtaining informed consent (mean rank = 1.74 for males and 1.57 for females, p = 0.003). The differences in perspectives on patients' rights are summarized in Table 6.

**Table 6:** Mann Whitney U test results for gender differences in perspectives on patients' rights

Variable	Gender	Freq	Mean rank	z	p-value
Every person in need of medical care is entitled to impartial access to health care.	Male	168	1.91	–	
	Female	122	1.97	2.07	0.039*
	Male	168	1.99	–	
The patient should be provided with appropriate medical services.	Female	124	1.99	0.32	0.748
	Male	163	1.87		
Patients should be represented in development of health policies.	Female	118	1.83	0.80	0.424
	Male	166	2.00		
Patients should receive empathetic and respectful care.	Female	125	1.99	1.15	0.249

				12	
Patients' participation in clinical trainings and programs should be voluntary.	Male	163	1.95	–	
	Female	125	1.99	1.98	0.047*
	Male	166	1.99	–	
Patients should have a right to privacy during clinical examination.	Female	125	1.99	0.33	0.736
	Male	163	1.98	–	
Patients should have a right to confidentiality.	Female	125	1.98	0.16	0.877
Patients should receive a full explanation of their cases and outcomes of care.	Male	165	1.99		
	Female	125	1.97	1.68	0.093
Patients or parent should sign informed consent forms before any medical procedure.	Male	164	1.96		
	Female	123	1.96	0.18	0.859
	Male	159	1.74		
A health provider may give medical treatment without informed consent.	Female	120	1.57	2.96	0.003*
	Male	164	1.93	–	
Patients can accept or refuse to participate in any medical research.	Female	124	1.94	0.16	0.872
	Male	167	1.98		
Patients should be treated in a hygienic environment.	Female	124	1.98	0.37	0.712
	Male	164	1.98		
Patients should be educated about precautions and financial costs.	Female	125	1.97	0.75	0.453
Every patient should be referred for a second opinion with or without request.	Male	153	1.79	–	
	Female	117	1.82	0.61	0.543
	Male	167	1.93		
A patient has the right to refuse or discontinue treatment.	Female	123	1.93	0.04	0.966
Patients should know the identity/name of the persons involved in health care.	Male	167	1.96		
	Female	124	1.92	1.65	0.098
	Male	167	1.88	–	
Patients should participate in care decision and choosing treatment plans.	Female	122	1.93	1.54	0.124
Every facility should designate a person or a committee for observance of patients' rights.	Male	162	1.95		
	Female	123	1.93	0.57	0.570

This table presents the summary of differences in perspectives on patients' rights using the Mann Whitney test.

Note:

* Significant at p<0.05

n=number of respondents

Differences in respondents' perspectives of patients' rights by academic program were analyzed using the Kruskal-Wallis test and are presented in Table 7. There were significant differences in perspectives on the patient's right to participate in care decisions and choose treatment plans across the three academic programs (mean rank = 1.98, 1.81, and 1.92 for BNS, BDS, and MBChB, respectively, p = 0.006). Considering the year of study, there were significant differences in perspectives on the patient's right to sign (or their parent to sign) informed consent forms before any medical procedure (mean rank =2.00, 1.98, 1.91, and 1.99 for Year 2, 3, 4 and 5 students respectively, p=0.029), and knowing the identity/name of the persons involved in health care (mean rank =1.75, 1.95, 1.98 and 1.94 for Year 2, 3, 4 and 5 students respectively, p=0.003).

**Table 7:** Kruskall Wallis test results for differences in perspectives on patients' rights by academic program

Variable	Academic program	Freq	Mean rank	*χ* ^2^	pvalue
Every person in need of medical care is entitled to impartial access to health care. The patient should be provided with appropriate medical services.	BSN	55	1.96	5.51	0.060
	BBDS	69	1.87		
	MBChB	166	1.95		
	BSN	55	2.00	0.72	0.695
	BDS	70	1.99		
Patients should be represented in development of health policies.	MBChB	167	1.99		
	BSN	53	1.79	2.52	0.284
	BDS	65	1.83		
	MBChB	163	1.88		

Patients should receive empathetic and respectful care.	BSN	55	2.00	0.74	0.69
	BDS	69	2.00		
	MBChB	167	1.99		

	BSN	55	1.95		
A patient has the right to refuse or discontinue treatment.	BDS	70	1.93	0.36	0.834
	MBChB	165	1.92		
	BSN	55	1.89		
Patients should know the identity/name of the persons involved in health care.	BDS	70	1.97	4.17	0.124
	MBChB	166	1.95		
	BSN	55	1.98		
Patients should participate in care decision and choosing treatment plans.	BDS	70	1.81	10.42	0.006*
	MBChB	164	1.92		
	BSN	54	1.96		
Every facility should designate a person or a committee for observance of patients' rights.	BDS	69	1.91		
	MBChB	162	1.95	1.74	0.418

Note:

* Significant at p<0.05

*χ*^2^ = Chi-square value

Differences in perspectives on patients' rights according to the three academic programs using the Chi-squared and Krus Wallis tests. The Krus Wallis test was used to analyse for differences among the three academic programs.

## Discussion

This study sought to examine health profession students' awareness and perspectives of patients' rights. Our findings suggest that most health profession students are aware of the PBR and are conversant with the different rights as stipulated in the Uganda Patient's Charter. Notably, there were differing opinions on the right to informed consent in medical care. In this study, 84% of the students were aware of the PBR, and this awareness is higher than what has been reported in the literature^9^. In a study among medical students and interns in Saudi Arabia reported an awareness of 69.3%. A similar Saudi Arabian study reported an awareness of 65.5% of the (4). Conversely, some studies have reported very low awareness of the PBR, with many students unfamiliar with the patient rights^2^,^10^.

Studies among health profession students report lectures as the main source of information on the PBR^4^,^9^. Most students in our study reported having learned about the PBR from lectures and the Internet. Notably, all students at MakCHS undertake a mandatory course on Ethics and Professionalism in year 1, where they are taught the principles of biomedical ethics, including patients' rights and responsibilities. Considering that most students cited the classroom as the means through which they learned about the PBR, it is plausible to attribute the high level of awareness to lectures. Therefore, it is key that students are exposed to knowledge of patients' rights early in their academic programs before exposure to patients in hospital wards.

Students' awareness of the existence of PBR is not enough; they should be encouraged to practice them. However, certain circumstances may not support this. For example, in 2020, a bill on patients' rights and responsibilities was tabled in the Uganda Parliament. However, the Minister of Health contended that enacting such a law would be detrimental to health service provision in the country^14^. She contended that it would increase the risk of litigation and result in abscondment from duty. Such a political position may contribute to unethical conduct and abuse of patients' rights. Indeed, some studies show that many health professionals do not observe patients' rights^6^,^15^. There is a need for proper guidance to health profession students on the importance of treating patients with dignity and respecting their rights. Students agreed on most patients' rights however, there were divergent opinions on the right to informed consent in medical care. Only two-thirds of respondents opined that patients should sign an informed consent form before any medical procedure. At the same time, another 64% agreed that a health provider may give treatment without informed consent. This is a bit surprising because all respondents were already exposed to hospital ward patients and had undertaken a course unit on Ethics and Professionalism in year 1. It seems some students did not appreciate the importance of soliciting informed consent. This could be attributed to the paternalistic approach to medical care in much of Africa^16^,^17^. Paternalism refers to the intentional overriding of the person's preferences. In this case, the physician justifies this action by making decisions that are deemed to be in the patient's best interest^18^.

Thus, in practice, the doctor utilizes his skills to choose the necessary interventions and treatment options most suitable for the patients without providing information to the patient to facilitate informed decisions. The right to self-determination is espoused in the World Medical Association Declaration of Helsinki^19^ and the Hippocratic Oath. Informed consent is premised on the ethical principle of autonomy and is a widely accepted ethical, legal, and regulatory requirement in medical care and research. Autonomy recognizes a person's right to hold views, make choices, and act based on their values and beliefs^18^,^20^-^22^. However, consent practices vary in context and reality from the theoretical ideals^23^. Similar to research, the informed consent form design necessitates detail about the risks, benefits, rights, and responsibilities of the patient and the physician, optimal for patient understanding based on their literacy level^24^. However, obtaining ideal informed consent may not be entirely possible in over-burdened and severely constrained health systems such as those in much of sub-Saharan Africa^25^. Another possible explanation for the differing views could be the reported questionable consent practices in clinical care in Uganda^25^-^31^. It reported that violations of patients' rights, discrimination, and unethical practices during patient care were rampant at the biggest university teaching hospital in Uganda. Notably, the healthcare providers who abuse patients' rights are the same ones who teach, supervise, and mentor these students. A disconnect between what students are taught about ethical practice and the protection of patients' rights and what they witness in clinical settings may impact their observance of patients' rights in their future practice^32^,^33^. Therefore, clinicians ought to be exemplary because oftentimes, students' and mentees' practices are influenced by their supervisors/mentors^34^.

Respondents offered differing responses when asked whether a patient's physical or mental state affects their ability to provide consent. The majority (66%) stated that a patient's physical or mental state affects their ability to provide consent, while 25% indicated that it does not, and 9% did not know. There is a belief that persons with mental illness cannot provide free and informed because their current mental state may interfere with their decision-making capacity^35^. Beauchamp and Childress^18^ set forth four elements of informed consent, including competence, adequate disclosure of information, understanding, and voluntariness of the final decision to consent. Competence usually denotes a person's ability to consent or refuse medical care, to be discharged against medical advice, or a person's capacity to live independently^36^. A patient with mental illness is considered incompetent when his/her ability to understand and make a sound judgment is questionable, but the presence of a mental illness alone does not equate to incompetence^36^. According to the Patient Rights and Responsibility Charter, medical care can be provided without consent if the patient cannot consent due to physical or mental incapacitation. The other grounds for providing care without informed consent include where it is impossible to obtain proxy consent, where the patient is a minor or incapacitated, where the patient has a disease of public health concern, and when the court orders^37^. Some aspects of these exceptions to waiver of informed consent are ambiguous, and this may be the reason for the varied responses. Religious affiliation was significantly associated with awareness of patients' rights. This finding suggests that students who identify with certain religious affiliations may have a higher level of awareness of patients' rights. The influence of religion on students' awareness of patients' rights has equally been reported by other authors. For example Abolfazli and Ataei^38^ reported the positive influence of Quranic teaching on enhancing the application and stabilization of the ethical and professional codes of conduct. Islam obligates the protection of confidential information of patients^11^. The Christian perspective emphasizes the therapeutic responsibility of the physician, the protection of patients' rights, and respect for life^39^. The Christian perspective also guides on helping patients make informed decisions by providing them with truthful information. The Christian perspective on the principle of autonomy accentuates the respect for patients' spirituality and religious beliefs, especially regarding issues such as the refusal of treatment^39^. In this study, we have no plausible explanation for the influence of religion on students' perspectives on patient rights.

What is needed is a qualitative study to get a deeper understanding of the influence of religion on clinical practice in these settings. There were significant gender differences in the patients' right to impartial access to health care (p= 0.039) and voluntary participation in clinical training (p= 0.047). These gender differences may be attributed to female students being generally more sensitive, emotional, and empathetic when it comes to providing health care to patients^40^,^41^. Studies have also shown that more women than men have engaged in and promoted patient-centered care that encourages the active involvement of patients in decision-making^41^. Female students may pay more attention to patients' rights details and wellbeing. Regarding the voluntary participation of patients in clinical training programs, we posit that some students think that all patients in teaching hospitals are obliged to participate in teaching activities. This may be ascribed to ignorance and the lack of awareness of the responsibilities of the patient in health profession education^42^. There have been calls for more training of health profession students and faculty members in the ethical involvement of patients in health profession education, in addition to better regulation of patients' involvement in students' learning^43^-^45^.

### Strengths and weaknesses

The main strength of the study was the diverse representation from different academic programs and years of study. Furthermore, all participants were exposed to patients and actively participated in patient care and learning in the hospital wards. The main weakness of the study was the recruitment of students from one academic institution, and the use of non-probability sampling. As such, the findings of the study may not be generalizable to undergraduate health profession students in Uganda. We acknowledge the weaknesses of non-probability sampling. The best approach would have been stratified random sampling, to ensure that all academic programs and years are fairly represented. However, it was logistically difficult to use this approach because clinical students are attached to different departments that are situated in different locations. Further, there were financial constraints given that this was master's student dissertation research. However, we tried to ensure fair representation of all programs and academic years by stratifying the study population as observed in Table 1.

## Conclusion

Most students were aware of the PBR and were conversant with the different patient rights prescribed therein. However, they seemed not to appreciate the patient's right to informed consent in medical care, as evidenced by the differing views they had. They also had limited awareness of the grounds for providing medical care without informed consent. There is a need for ongoing education and training of health profession students on patients' rights and responsibilities to enhance their awareness and understanding. It is imperative that they receive comprehensive education on the prioritization of patients' rights and dignity, informed consent, as well as fostering open and effective communication with patients. Health profession students should also be encouraged to respect patients' rights in training and clinical practice when they eventually become health professionals. Qualitative research should also be conducted to get a deeper understanding of facilitators and barriers to the observance of patient rights in medical care.

## Data Availability

The research data for this study are freely available at 10.6084/m9.figshare.27021826.
